# By the Book or Out of the Box? Top Decision Maker Cognitive Style, Gender, and Firm Absorptive Capacity

**DOI:** 10.3389/fpsyg.2021.622493

**Published:** 2021-01-28

**Authors:** Christopher Pryor, Robert Hirth, Yanghua Jin

**Affiliations:** ^1^Warrington College of Business, University of Florida, Gainesville, FL, United States; ^2^Earl N. Phillips School of Business, High Point University, High Point, NC, United States; ^3^School of Business Administration, Zhejiang Gongshang University, Hangzhou, China

**Keywords:** absorptive capacity, cognitive style, adaptive-innovative theory, microfoundations, opportunity exploitation

## Abstract

Despite scholars’ early emphasis on the role people play in fostering firms’ absorptive capacity (AC), research has not deeply explored the individual-level antecedents of this important capability. We draw on adaptive-innovative theory to explain how top decision makers’ cognitive styles can influence the degree to which their firms develop AC. Top decision makers who have high *adaptive cognitive style* prefer to adhere to existing norms, follow established procedures, and rely on current knowledge, and we argue that these attributes will strengthen those dimensions of AC based on firms’ existing knowledge and knowledge-assimilation abilities. Top decision makers who have high *innovative cognitive style* are more likely to reframe information, experiment with new problem-solving approaches, and take risks by violating norms, and we argue that these attributes may strengthen those dimensions of AC based on firms’ acquisition of new knowledge and the assimilation of knowledge throughout the firm. We also argue that gender differences may moderate these effects. Empirical results support our hypotheses.

## Introduction

Absorptive capacity (AC) refers to a firm’s ability to absorb new knowledge from the external environment, retain and disseminate the knowledge throughout the firm, and use the knowledge to create and capture customer value (Cohen and Levinthal, 1990; Zahra and George, 2002). Researchers have found that AC enables firms to respond to changing environmental and competitive conditions (Jansen et al., 2005), perceive and understand new knowledge as it enters the firm (Cohen and Levinthal, 1990), and share knowledge among units and members of the firm (Chang et al., 2012). By unlocking these knowledge-related advantages, AC can strengthen firms’ ability to produce new products and services (Nooteboom et al., 2007) and improve performance (Patel et al., 2015). For these reasons, researchers have argued that AC is an important component of firms’ ability to identify and exploit new opportunities *via* entrepreneurship (e.g., Zahra et al., 2009), given the importance of new knowledge acquisition and assimilation to identifying new market opportunities (Tang et al., 2012). As Ireland et al. (2003, p. 975) write: a “firm’s AC is linked to the effective use of (strategic entrepreneurship).”

One key antecedent of firms’ AC are people who possess the capacity to absorb and process new information. The earliest conceptualizations of AC emphasized the role played by individual people in a firm in fostering firm-level AC (Cohen and Levinthal, 1990). Nevertheless, despite the early emphasis placed on individuals, there has been a little empirical examination of how people in a firm cultivate AC (Volberda et al., 2010; Distel, 2019). Even less research has focused on the effect firms’ top decision makers may have on AC, despite the influence top decision makers have in setting firm strategy and guiding firm behaviors (Hambrick and Mason, 1984; Bromily and Rau, 2016; Wang et al., 2016). Cohen and Levinthal (1990, pp. 131–132) write: “An organization’s AC will depend on the absorptive capacities of its individual members … it also depends on transfers of knowledge across and within sub-units that may be quite removed from the original point of entry.” Given that, few people in a firm may contribute more to AC than the top decision maker, who serves as the cognitive nexus of a firm (Helfat and Peteraf, 2015), transferring valuable parcels of knowledge across their firms’ units. The lack of research focusing on the relationship between top decision makers and firms’ AC constitutes a severe limitation because, while research has acknowledged the vital role people play as repositories of information in a firm (Kogut and Zander, 1992), as well as the influence that top decision makers have in promoting and sharing knowledge in a firm (Arendt et al., 2005), our understanding of how these mechanisms may similarly promote AC is much weaker.

**Figure 1:**
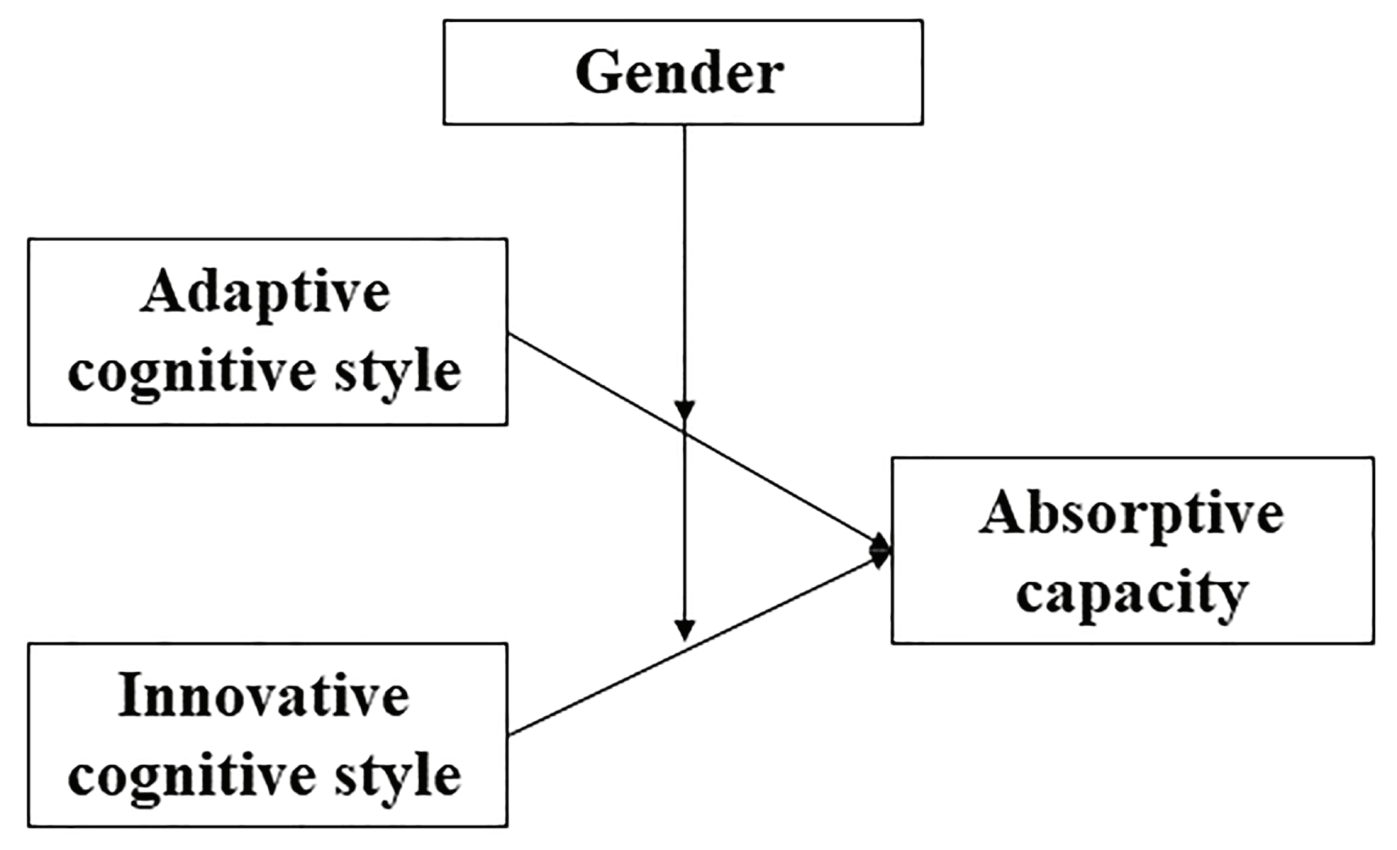
Top decision makers’ cognitive style, absorptive capacity, and opportunity exploitation.

This paper addresses this limitation by focusing on the relationship between the heterogeneous characteristics of firms’ top decision makers and their firms’ AC. We draw on Kirton’s (1976, 2004) adaption-innovation theory to propose that top decision makers’ cognitive style can influence firm-level AC. Adaption-innovation theory proposes that when encountering a problem, people’s preferences for contending with the problem fall on a continuum between adaption, in which conservative tried-and-true methods are used to adhere to normative guidelines, and innovation, in which existing methodologies are cast aside to produce solutions that may not meet expectations (Buttner and Gryskiewicz, 1993). As shown in Figure 1, we argue that top decision makers’ adaptive cognitive style and innovative cognitive style are positively related to firms’ AC. Adaptive cognitive style corresponds to the reliance of existing knowledge structures and processes, which can facilitate the integration of new knowledge into a firm. Innovative cognitive style corresponds to the pursuit of new methods and new information, facilitating the search for new knowledge and the integration of new knowledge across people and divisions within firms. Adaption-innovation theory may be a particularly useful lens through which to view the top-decision-maker-AC relationship because AC research highlights the importance of leveraging stocks of existing knowledge to acquire, integrate, and use new knowledge (Zahra and George, 2002; Nagle and Teodoridis, 2020). Similarly, the underlying adaption-innovation theory is an assumption regarding how people prefer to handle knowledge. When driven by an adaptive cognitive style, people prefer to access existing knowledge and strengthen current capabilities while an innovative cognitive style leads people to pursue new knowledge that can develop new capabilities (Chilton and Bloodgood, 2010). Therefore, top decision makers’ cognitive styles may directly bear on their firms’ AC.

Top decision makers’ cognitive styles may affect the degree to which their firms exhibit AC, and we further propose that this effect is moderated by top decision makers’ gender. Differences have been found to exist between men and women in terms of how they lead their firms (Ingersoll et al., 2019). In particular, researchers have found that women tend to pursue innovative projects more vigorously while also adhering more strongly to established rules than men (Glass et al., 2016). Drawing on insights from this literature – together with research that describes important gender differences in cognitive abilities – we propose that the relationship between top decision makers’ cognitive style and AC will be stronger for women than for men (see Figure 1) because women will tend to place greater emphasis on routine and have advantages related to verbal ability, which we argue can enhance their ability to promote AC. We also expect that the relationship between top decision makers’ innovative cognitive style and AC will be stronger for women because they tend to lead innovative efforts and build cohesive teams around shared ideas.

This study contributes to research on AC, top decision makers, and firm microfoundations. First, Volberda et al. (2010) called for greater research on the individual-level antecedents of firm-level AC, and while there has been significant development of our understanding of how and why firms may develop AC, relatively less progress has been made concerning individual-level attributes [with notable exceptions (e.g., Distel, 2019)]. Therefore, our paper, which focuses on the effects that top decision makers’ cognitive style may have on AC helps resolve the inherent incompleteness in AC research (Volberda et al., 2010). Second, research on top decision makers has begun to pay increasing attention to attributes of top decision makers that are not easily observable, such as personality, cognition, and motivations, whereas earlier research focused on age, tenure, or education (e.g., Wang et al., 2016). Extending research into top decision makers’ cognitive attributes is important because it can develop our understanding of the cognitive mechanisms by which top decision makers perceive and respond to their firms’ environment (Neeley et al., 2020). Therefore, our study contributes to this research stream by focusing on a psychological attribute, cognitive style, that can affect AC. At the same time, management research has explored gender differences in terms of leadership (e.g., Burke and Collins, 2001), compensation (e.g., Gupta et al., 2015), board interactions (e.g., Post and Byron, 2015), and career advancement (e.g., Bonet et al., 2020). However, little research has focused on the cognitive attributes that can differ across genders and how these differences influence firm outcomes, which is an important topic to consider, given the influence top decision makers’ cognitions wield as they lead their firms (Graf-Vlachy et al., 2020). Our empirical findings suggest that women seem to be better able to leverage both adaptive and innovative cognitive styles to foster AC. This contribution may have theoretical and practical significance for scholars and firm stakeholders who are interested in understanding how firms may better pursue strategic entrepreneurship.

Finally, this paper contributes to microfoundations research by exploring the individual-level antecedents of AC, which is a firm-level capability, and strategic entrepreneurship more broadly. While scholars have called for a greater understanding of the micro-level influences macro-level outcomes in management (Felin et al., 2012) and despite theoretical arguments that firm-level AC is built upon individual people’s ability to acquire, absorb, and understand new knowledge (Cohen and Levinthal, 1990), we have little understanding regarding these influences as they concern AC (Distel, 2019). Therefore, our study contributes to microfoundations research by concentrating on the individual top decision maker and their cognitive style as an unexplored but influential antecedent of their firm’s AC.

This paper proceeds as follows. First, as background, we define and describe AC as well as explain adaptive-innovation theory. Drawing on this research, we next offer a set of hypotheses that link top decision makers’ cognitive styles with their firms’ AC. We then provide a description of our method and results. The paper closes with a discussion of our results, the implications our work may have for research, and the limitations of the study.

## Theory and Hypotheses

### Absorptive Capacity

Firms vary in their ability to launch innovative products and services, and scholars have suggested that some of this heterogeneity may be explained by firms’ ability to learn and use new information, known as AC (Cohen and Levinthal, 1990; Todorova and Durisin, 2007; Szulanski et al., 2016). AC has been conceptualized as a firm-level dynamic capability, which means that AC comprises one of the “organizational and strategic routines by which firms achieve new resource configurations as markets emerge, collide, split, evolve, and die” (Eisenhardt and Martin, 2000, p. 1107). AC itself is composed of sets of routines that enable firms to *acquire* new information from the external environment, *assimilate* that new information into firms as knowledge, and *transform* that knowledge into useful material that can be more easily used by people throughout the firm (Zahra and George, 2002; Lewin et al., 2011). Empirical research has found that firms’ AC positively contributes to their acquisition of new knowledge, development of new products and services, and financial performance (Song et al., 2018; Xie et al., 2018). Below, we discuss each of these categories of AC routines and describe how firms’ top decision makers can influence their enactment. As Cohen and Levinthal (1990, p. 132) note, “[AC] depends on the individuals who stand at the interface of either the firm and the external environment or at the interface between the subunits within the firm.” Hence, our focus on top decision makers, who wield significant influence in their firms and act as the conduit by which information passes across different units of their firms (Hambrick and Mason, 1984; Helfat and Peteraf, 2015).

#### Acquisition

The acquisition of new information from the external environment is the first component of AC. In order to respond to the competitive environment or to perceive and respond to changing customer demands, firms can collect information from the environment (Yli-Renko et al., 2001). The processes by which firms gather information from their external environments can be routinized, and firms that have a distinctive capability at acquiring new information can include those that incentivize employees throughout the firm to regularly monitor the environment, that experiment and learn from feedback, or that encourage employees to share ideas with peers in the industry (e.g., Spencer, 2003). Top decision makers can influence their firms’ information acquisition efforts, such as by modeling the behaviors themselves or by creating and enforcing incentives that compel employees to scan the external environment (Pryor et al., 2019). Top decision makers can also control the intensity of their firms’ R&D, which is an important antecedent of information acquisition (Cohen and Levinthal, 1990), as firms that need to deploy new products and services will tend to be those more likely to need updated information regarding changing environmental conditions.

#### Assimilation

The second component of firms’ AC – their ability to assimilate new information – depends upon firms’ prior knowledge, as well as the processes they have in place to integrate new information into their existing knowledge structures. Cohen and Levinthal (1990) emphasized the role of prior knowledge as part of the foundation of AC: prior knowledge helps firms interpret new information, it is the raw material of their current R&D activities, and it is the basis of their innovative capability (Cohen and Levinthal, 1990). Prior knowledge helps firms understand whether new knowledge is valuable and relevant, and information in the environment that is unrelated to firms’ current knowledge base is more likely to pass unrecognized or unvalued (Lane and Lubatkin, 1998; Song et al., 2018).

Possessing the capability to integrate new information can also be critical. Considering that more valuable new information is often significantly unlike existing knowledge and that diverse information sources are more useful than more homogeneous sources (Cohen and Levinthal, 1990), integration can be particularly challenging. Research has found that firms that are more adept at integrating new information experience greater improvement in innovative outputs (Wang et al., 2018). Firms that are adept at integrating new information may be those that have developed routines related to articulating new information, regularly conferring with leaders and employees throughout the firm about the implications of the new information, and deliberately finding connections, or associations, between new information and existing knowledge (e.g., Zahra et al., 2007).

Top decision makers can influence the prior knowledge possessed by their firms, such as by investing (or not) in acquiring and developing human capital (Wang et al., 2020). They can also help foster culture that encourages knowledge sharing and feedback-seeking, which can further enhance firms’ stocks of prior knowledge (Bendig et al., 2018). Additionally, because top decision makers act as boundary spanners – between the firm and the external environment, between the firm and its competitors, and between the various operational divisions within their firms (Hambrick and Mason, 1984; Liu et al., 2018) – they play a crucial part in how new knowledge is integrated within the firm. For instance, because top decision makers occupy the top position in a hierarchy, they have the unique ability (relative to sub-level managers or employees) to create management roles that are responsible for sharing information between departments (e.g., Lewin et al., 2011).

#### Transformation

Zahra and George (2002, p. 190) explain that transformation “yields new insights, facilitates the recognition of opportunities, and at the same time, alters the way the firm sees itself and its competitive landscape.” Through transformation, firms use assimilated new information to update routines, create new knowledge, and develop new ideas for products and services (Todorova and Durisin, 2007). Firms competent at transformation can include those that have developed routines that enable employees to use new knowledge or to practice new skills, such as allowing employees to have time each week to work on their own projects, reflect on personal or team progress, or encouraging risk-taking (e.g., Lewin et al., 2011). As with the preceding components of AC, it can be likely that top decision makers can also shape the degree to which their firms exhibit transformation. For instance, top decision makers can encourage employees and managers to pursue innovations through incentives or by personally modeling innovative or risk-taking behaviors (e.g., Wang and Das, 2017). Top decision makers might also encourage their employees to collaborate with creative or scientific experts outside the firm (Makri and Scandura, 2010).

### Adaption-Innovation Theory

When people encounter problems, they tend to exhibit differences in how they prefer to understand the problem and develop a solution. These preferences are a type of cognitive style, which concern how people approach and process information (Carnabuci and Dioszegi, 2015). While early research led to the development of many different types of cognitive styles, a more recent trend has been toward a coalescence around the notion that people may exhibit either a cognitive style that is convergent, analytical, detail-oriented, and adheres to existing knowledge structures (i.e., adaptive), or a cognitive style that is divergent, intuitive, and impulsive (i.e., innovative; Kozhevnikov, 2007). Subsequent research using Kirton’s (1976) adaptive-innovative theory has found empirical support for these cognitive styles (Shalley et al., 2004), suggesting that people may tend to exhibit one style more strongly than the others (Payne, 1987). However, these styles are conceptualized independent from the other, which means, in theory, a person may exhibit both styles when encountering a problem (Taylor, 1989), and studies typically theorize and measure these styles using independent scales (e.g., Miron-Spektor et al., 2011).

People who exhibit an adaptive cognitive style tend to prefer structure, procedure, sequence, and order (Chilton and Bloodgood, 2010). They tend to rely on existing sources of information and provide solutions that have been proscribed by supervisors, teachers, or other social norms (Carnabuci and Dioszegi, 2015). They are meticulous and perceive significant risk in breaking from established procedures (Kwang and Rodrigues, 2002). People who exhibit the innovative cognitive style tend to treat problems as opportunities to try new ideas, reframe existing information, and produce a high quantity of solutions, which may or may not solve the problem they face (Carnabuci and Dioszegi, 2015). They are also not characterized by the same hesitancy to break social norms or the expectations of others or take the associated risks of trying new methods of problem-solving (Shalley et al., 2004).

### Adaptive Top Decision Makers and Absorptive Capacity

We believe that top decision makers’ adaptive cognitive style will be positively related to firms’ AC for at least three reasons. First, top decision makers with an adaptive cognitive style will tend to devote more effort to reinforcing existing patterns of behavior in their firms, including the routines that underly firms’ AC (Zahra and George, 2002). Routines are a useful organizational resource because they help people in firms reduce uncertainty, obtain behavioral efficiencies, and avoid conflict (Wilhelm et al., 2015). Scholars have argued that people with high adaptive cognitive style prefer work that is routine and predictable (Baer et al., 2003). Therefore, we expect that top decision makers who possess a high adaptive cognitive style will similarly prefer organizational interactions and behaviors to be routine, which can strengthen the routines that constitute all three of the components that constitute firms’ AC, *acquisition*, *assimilation*, and *transformation*.

Second, the top decision maker with an adaptive cognitive style will tend to be an associative thinker (Jabri, 1991), which means they will rely on existing knowledge to frame new information, develop incremental solutions to existing problems, and seek to reinforce their current understanding with updates as opposed to completely changing their perspective (Scott and Bruce, 1998). Association – or the tendency to link new information to existing knowledge – is a key element of AC, and it is particularly emphasized in Cohen and Levinthal (1990), who describe associative thinking as important to building stocks of prior knowledge, which constitutes the *assimilation* component of AC, and developing new ways of understanding information, which constitutes the *transformation* component of AC. Furthermore, Lowik et al. (2017) linked adaptive cognitive style with associative thinking and argued that people who exhibit this tendency also tend to be better communicators and competent at articulating information. Such top decision makers, who can make associative connections between new information and existing knowledge and who can articulate and communicate these thoughts to others in the firm may enhance their firm’s *assimilation* of knowledge, strengthening AC.

Third, top decision makers with an adaptive cognitive style will prefer to work with explicit knowledge, as opposed to tacit knowledge (Chilton and Bloodgood, 2010). When exhibited by top decision makers, who can influence the acquisition and retention of firms’ stock of knowledge (Bendig et al., 2018), a preference for explicit information could lead to a greater overall level of codification. The codification of information or knowledge has been empirically linked to higher rates of organizational learning (e.g., Principe and Tell, 2001) – benefiting firms’ prior knowledge and, thus, *assimilation* – and to higher rates of innovation (e.g., Sorensen and Lundh-Snis, 2001), in turn, strengthening *acquisition* and *transformation* (Song et al., 2018). As a further consequence, of the preference for explicit knowledge, which can foster codification, firms may exhibit stronger organizational memory (Fiedler and Welpe, 2010). Consistent with Cohen and Levinthal (1990), greater memory constitutes prior knowledge, which is an important building block of AC, and greater memory can also facilitate the *assimilation* of new information because the firms’ existing knowledge is easier to access. In sum, we believe that top decision makers who exhibit an adaptive cognitive style will promote AC in their firms due to their preference for routinization, their tendency for associative thinking, and their inclination for explicit, codified knowledge. Therefore, we hypothesize:

*Hypothesis 1*: Top decision makers’ adaptive cognitive style will be positively related to their firms’ AC.

### Innovative Top Decision Makers and Absorptive Capacity

We believe that top decision makers’ innovative cognitive style will be positively related to their firms’ AC for at least three reasons. First, innovative top decision makers who exhibit an innovative cognitive style practice and promote divergent thinking, creativity, associating previously unconnected information, reframing information, and producing many possible solutions, not all of which conform to expectations (Gong et al., 2013; Carnabuci and Dioszegi, 2015). These tendencies may also contribute to firms’ *acquisition* of new information. Zahra and George (2002) explain how *acquisition* in AC is propelled by firms’ effort to acquire new information. This is one reason R&D has been portrayed, at least partly, as an antecedent of AC – the more innovative efforts a firm undertakes, the more it will need new information to fuel those efforts (Cohen and Levinthal, 1990; Song et al., 2018). Therefore, we believe that top decision makers who have an innovative cognitive style will likely lead their firms to pursue more innovative products and services (e.g., Emsley et al., 2006), which can, in turn, drive their firms to dedicate more effort to acquire new knowledge, leading to greater AC.

Second, top decision makers with an innovative cognitive style may have a greater willingness to unlearn outdated or useless information (Lowik et al., 2017). Unlearning, which refers to discarding information (Akgun et al., 2003), has been described as an important element of AC because when firms unlearn prior information, they may more readily *assimilate* new information as well as develop new products or other operational methods (Wensley and Navarro, 2015). Unlearning can be especially critical when the information firms acquire new information that contradicts or is widely divergent from firms’ existing knowledge – to be able to use this information, firms must be willing to abandon old ideas (Cepeda-Carrion et al., 2012). Top decision makers, therefore, who have an innovative cognitive style and who are willing to unlearn prior knowledge may contribute to firms’ ability to *assimilate* new information and to *transform* based on the newly absorbed knowledge.

Third, top decision makers with an innovative cognitive style may enhance their firms’ ability to *transform* because of their greater willingness to create imaginative new combinations with information and experiment with unexpected ideas (Carnabuci and Dioszegi, 2015; Lowik et al., 2017). These top decision makers’ tendency toward bisociative thinking (Mudd, 1995) complements the task of *transformation* in which “two apparently incongruous sets of information [are combined] … to arrive at anew schema” (Zahra and George, 2002, p. 190). At the same time, top decision makers who have innovative cognitive styles tend to solicit feedback on their creative ideas more frequently across a broader number of people (De Stobbeleir et al., 2011). This way, top decision makers with an innovative cognitive style are personally more creative, but they are also more likely to spread their ideas throughout the firm, both of which can foster even higher levels of creativity and strengthening the *transformation* component of their firms’ AC. In sum, we believe that top decision makers with innovative cognitive styles are more likely to drive their firms to expend more effort to *acquire* new information, have a greater willingness to unlearn old information and thus improve *assimilation*, and have a greater capacity to create innovative ideas and spread those ideas throughout the firm and boost *transformation*. Therefore, we hypothesize:

*Hypothesis 2*: Top decision makers’ innovative cognitive style will be positively related to their firms’ AC.

### Gender Differences: Cognitive Styles and Absorptive Capacity

As top decision makers, men and women may differ in ways that are relevant to firms’ AC. In this section, we draw on research concerning gender differences in cognition, as well as research that has explored the differences between men and women top decision makers to describe why both of the effects we describe above will tend to be stronger for top decision makers who are women than for top decision makers who are men.

We expect that the positive relationship between top decision makers’ adaptive cognitive style and firms’ AC will be stronger for female top decision makers relative to male top decision makers. Earlier, we had argued that the adaptive cognitive style would lead top decision makers to prefer routinization, associative thinking, and explicit knowledge. The empirical record suggests that women who have an adaptive cognitive style may have at least two advantages over men who have an adaptive cognitive style in terms of stimulating firm-level AC. First, women tend to exhibit less impulsiveness than men, which means they are more likely to plan, follow procedures, and deliberate before acting (Moffitt et al., 2001; Szabo and Jones, 2019). This difference could contribute to female top decision makers’ ability to foster and maintain routines in their firm, such as the routines that undergird AC. In contrast, top decision makers who are more impulsive, which tends to characterize males, may not engage in the same planning, deliberative action, and adherence to procedures. As a consequence, their firms may develop weaker routines, which could hamper their firms’ development of AC or lead to the episodic manifestation of AC, such as when the firm encounters an acute competitive threat or perceives an environmental shock. Relatedly, there is evidence that women have a greater capacity for mindfulness, defined as “being attentive to and aware of what is taking place in the present” (Brown and Ryan, 2003, p. 822), due to in part to brain structure and their ability to simultaneously employ both brain hemispheres (Shao and Skarlicki, 2009). Mindfulness has been connected to firms’ successful management of renewed routines (Salvato, 2009), which could indicate that female top decision makers, when coupled with an adaptive cognitive style, may be especially adept at enacting routines in their firm, which could cultivate AC.

Second, adaptive top decision makers prefer to work with explicit knowledge (Chilton and Bloodgood, 2010), and we argue above that such top decision makers may increase the amount of codified knowledge in the firm, which could enhance all three aspects of AC. Among the more consistent research findings concerning gender differences and cognition is the consistent and lifelong advantage women have in verbal ability (Asperholm et al., 2019). Verbal ability includes language fluency, word recall, and speaking (e.g., Wallentin, 2009). When coupled with improved verbal ability, top decision makers with an adaptive cognitive style could be more successful at codifying knowledge in their firm. Because women have the advantage concerning verbal ability, we believe this will contribute to strengthening the positive relationship between adaptive cognitive style and AC. Therefore, we hypothesize:

*Hypothesis 3*: Top decision maker gender will moderate the effect between adaptive cognitive style and AC such that the relationship will be stronger for female top decision makers than for male top decision makers.

We also expect that the relationship between top decision makers’ innovative cognitive style and their firms’ AC will be stronger for female top decision makers than for male top decision makers. The innovative cognitive style in top decision makers leads them to expend more effort to acquire new information, a greater willingness to unlearn prior knowledge, and a tendency to develop more innovative ideas and spread those ideas throughout the firm. An increasing amount of research has argued that when it comes to occupying firms’ top executive positions, women and men’s experience during career ascension can subsequently influence how they lead their firms (Fitzsimmons et al., 2014). We believe that these unique experiences may contribute to female top decision makers’ ability to wield an innovative cognitive style and contribute to AC. In particular, because so few women occupy firms’ upper echelons, women who do ascend to the top are subject to added scrutiny and can suffer from delegitimization (Post et al., in press). In response, women may attempt to develop “novel strategic vision around which they develop collective support” (Bowles, 2012, p. 195), and they feel pressure to demonstrate capabilities in excess of what their male peers may achieve (Glass and Cook, 2020). Empirical evidence lends support to the notion that greater female representation among firms’ upper echelons can increase the effort devoted toward innovation (Chen et al., 2018; Post et al., in press). Because women top decision makers may devote more effort to innovative initiatives than their male counterparts – and when coupled with top decision makers’ innovative cognitive style – firms may subsequently engage in a greater amount of information acquisition.

At the same time women top decision makers may be more likely to lead innovative efforts, they are also more likely to work with others in the firm, sharing ideas, obtaining feedback, and building coalitions around their change efforts (e.g., Zhang et al., 2015). By involving more people in the decision making process, obtaining feedback on their ideas, and sharing information throughout the firm, female top decision makers may create a higher degree of intra-firm communication (e.g., Eisenberg et al., 2019). Thus, by improving communication in a firm, knowledge can more efficiently travel between firms’ members, which can increase both the degree to which information is assimilated into the firm, as well as how well people in a firm can take new information and transform the firm functions and outputs (Zahra and George, 2002). For these reasons, we believe that female top decision makers with an innovative cognitive style may be better enabled – through their preference for innovative change efforts and a tendency to interact with others and improve communication – to develop stronger firm-level AC than their male counterparts. Therefore, we hypothesize:

*Hypothesis 4*: Top decision maker gender will moderate the effect between innovative cognitive style and AC such that the relationship will be stronger for female top decision makers than for male top decision makers.

## Method

### Sample and Procedure

To test our hypotheses, we relied on a sample of top decision makers drawn from an alumni database of a public university in the southern United States. The database included the job titles for each person, and we selected participants who had job titles indicating they were the top decision makers in their firms, such as president, founder, CEO, director, or partner. This selection criteria led to the creation of a list with 2,468 names. Because we used mailed paper surveys to collect data, it was important for us to attempt to mitigate common method bias concerns, and we did so by employing temporal separation in data collection (Podsakoff et al., 2003), which meant separating our survey measures across two phases of data collection, with 2months separating each phase. The first phase of surveys contained measures used as control variables and for variables that measured participants’ adaptive and innovative cognitive styles. The second phase of surveys included the measure for firms’ AC.

In April 2011, the first phase of surveys was sent by mail to all 2,468 participants identified from the alumni database. There were 456 responses, and these participants received the second phase surveys. There were 265 responses to the second phase, representing an overall response rate of 10.7%. This response rate is consistent with response rates obtained in similar survey-based studies of top decision makers (e.g., Boone and Hendricks, 2009).^1^ Of the top decision makers who completed both surveys, their average age was 55.86 (SD = 10.92) and about 15% were women. About 55% had earned a bachelor’s degree, 33% a master’s degree, about 7% had earned a post-graduate degree, and the remaining 5% had not earned a bachelor’s degree. Top decision makers in the sample represented firms that were about 31.17years old (SD = 27.94) at the time the surveys were completed and employed about 198 people (SD = 1,540.57). The industries represented in the sample included consumer services, consulting and other business services, engineering and construction firms, and manufacturing and energy production firms. No differences were found in terms of age, gender, or education between top decision makers that responded to both survey phases to those who only responded to the first phase, nor did firms represented by top decision makers in the final sample differ in terms of age or size from those represented by top decision makers who responded only to the first survey phase.

### Measures

#### Cognitive Style

We measured top decision makers’ cognitive styles with items developed by Jabri (1991) and using 7-point Likert-type scales (1 = strongly disagree to 7 = strongly agree). For adaptive cognitive style, we used 10 items, including, “I enjoy or prefer adhering to commonly established rules of my work,” “I enjoy or prefer paying strict regard to the sequence of steps needed for the completion of a job,” and “I enjoy or prefer being methodical and consistent in the way I tackle problems.” The Cronbach alpha for this measure was 0.86. Eight items were used to measure innovative cognitive style, including “I enjoy or prefer being confronted with a maze of ideas which may, or may not, lead me somewhere,” “I enjoy or prefer making unusual connections between ideas even if they are trivial,” and “I enjoy or prefer searching for novel approaches not required at the time.” The Cronbach alpha for this measure was 0.75.

#### Absorptive Capacity

We measured firms’ AC with nine items developed by Cadiz et al. (2009). Participants responded *via* a 7-point Likert-type scale (1 = strongly disagree, 7 = strongly agree). Example items included “People in my firm are able to decipher the knowledge that will be most valuable to us,” “The shared knowledge within my firm makes it easy to understand new material presented within our technical areas,” and “New technical knowledge can be quickly applied to our work.” The Cronbach alpha for this measure was 0.87.

#### Top Decision Maker Gender

Top decision maker gender was assessed by participants’ response to the question “Are you a male or female?” (Male = 0, female = 1).

#### Controls

Controls were included for top decision makers’ age and education level. Age was included as a control because age is positively related to the acquisition of practical experience, and we expect that older top decision makers may make better choices and exhibit greater competence in their firms (e.g., Dragoni et al., 2011), which can promote their firms’ AC. We included the control for education because it can improve top decision makers’ cognitive abilities, which can influence firms’ AC (Lowik et al., 2017). We controlled for whether top decision makers were firm founders because prior research has linked founder status to greater levels of control, identification, and status in their firms, which can influence the degree to which and the reasons why they control firm behavior (Pryor et al., 2019). A control was also included for top decision makers’ executive experience (i.e., how many years they have been in their current position) for two reasons. First, top decision makers who have more extensive experience in their position could have had more time to influence firm-level outcomes relative to top decision makers who have less experience, and second, top decision makers who have been in their position relatively longer may feel less pressure to demonstrate their capability as leaders. Controls were included for firm size (i.e., number of employees) and firm age because research has linked these characteristics to innovative performance: smaller firms, which are less encumbered by bureaucratic structures, may be able to achieve greater innovative output due to their flexibility (Acs and Audretsch, 1987), whereas aging firms are similarly less able to accommodate new information and revise existing assumptions about the market, product, and competitive conditions (Sorensen and Stuart, 2000). We also included industry controls (three dichotomous industry variables: agriculture and energy production, 1 = yes, 0 = no; business-to-business services, 1 = yes, 0 = no; consumer-oriented services, 1 = yes, 0 = no) because industries may vary in terms of dynamism, which can reduce the importance of AC as a source of heterogeneity among firms (Xue et al., 2012; Larraneta et al., 2014).

## Results

We conducted a CFA for variables measured by participants’ response to scaled items (i.e., adaptive cognitive style, innovative cognitive style, and AC). Table 1 reports the results of these analyses, and the three-factor model shows adequate fit (Tucker-Lewis index = 0.90, confirmatory factor index = 0.91, standardized root mean residual = 0.08). Compared the three-factor model to the possible two-factor models demonstrates significantly better fit, indicating discriminant validity, and all item loadings were significant, indicating convergent validity. A correlation matrix with means and standard deviations for each variable is included in Table 2.

**Table 1:** Results of confirmatory factor analysis.

	*χ*^2^	*df*	TLI	CFI	SRMR
Three-factor model	537.28	321	0.90	0.91	0.08
Two-factor model (adaptive-innovative cognitive styles, absorptive capacity)	1166.39	323	0.63	0.66	0.12
Two-factor model (adaptive cognitive style – absorptive capacity, innovative cognitive style)	1480.23	323	0.50	0.53	0.14
Two-factor model (adaptive cognitive style, innovative cognitive style – absorptive capacity)	1173.28	323	0.63	0.66	0.12
One-factor model	1808.73	324	0.35	0.40	0.16

TLI = Tucker-Lewis index; CFI = comparative fit index; SRMR = standardized root mean square residual. *N* = 265.

**Table 2:** Means, standard deviations, and correlations.

Variable	Mean	Std. Dev.	1	2	3	4	5	6	7	8	9	10	11	12
1. Top decision maker age	55.86	10.92												
2. Top decision maker gender	0.15	0.36	−0.09											
3. Top decision maker education	5.36	0.87	0.11	−0.03										
4. Top decision maker founder status	0.69	0.46	0.15^*^	−0.08	0.09									
5. Top decision maker executive experience	16.62	10.64	0.50^**^	−0.12	−0.01	0.25^**^								
6. Firm size (number of employees)	197.81	1540.57	0.01	0.08	−0.03	−0.14^*^	−0.09							
7. Firm age	31.17	27.94	−0.15^*^	0.07	0.04	0.58^**^	−0.19^**^	−0.18^**^						
8. Industry (agriculture and energy production)	0.06	0.23	0.00	−0.01	−0.04	0.06	0.21^**^	−0.03	−0.02					
9. Industry (business-to-business services)	0.49	0.50	0.02	−0.10	−0.04	−0.07	−0.04	0.09	0.01	−0.24^**^				
10. Industry (consumer-oriented services)	0.46	0.50	−0.02	0.11	0.06	0.05	−0.06	−0.07	0.00	−0.22^**^	−0.89^**^			
11. Adaptive cognitive style	4.92	0.94	0.17^**^	0.11	−0.05	0.13^*^	0.04	−0.03	0.08	−0.13^*^	0.00	0.06		
12. Innovative cognitive style	5.16	0.72	0.00	−0.01	−0.01	0.11	0.01	0.05	0.15^*^	0.00	−0.05	0.05	−0.06	
13. Absorptive capacity	5.19	0.85	0.13^*^	0.07	0.05	0.13^*^	0.04	−0.05	0.07	−0.05	0.09	−0.07	0.26^**^	0.13^*^

* *p* < 0.05;

** *p* < 0.01;

*N* = 265.

**Figure 2:**
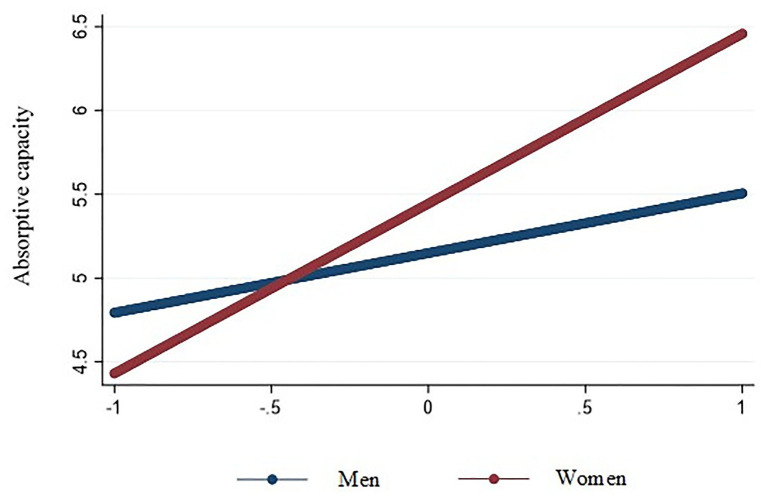
Interaction of top decision makers’ adaptive cognitive style and gender on absorptive capacity.

**Figure 3:**
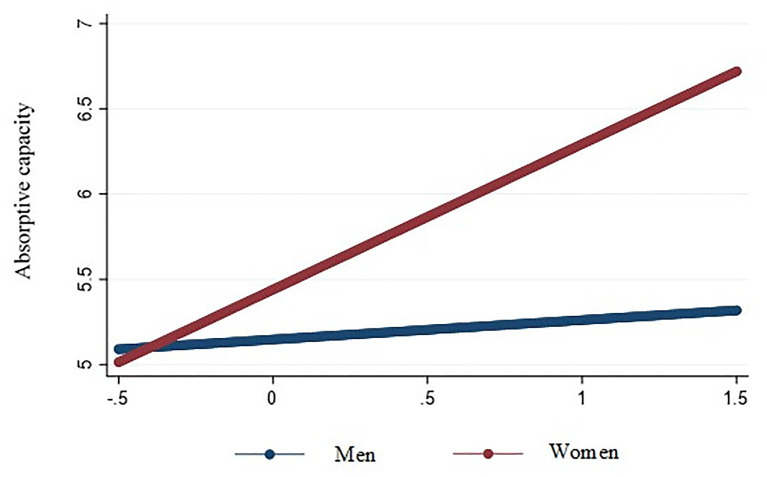
Interaction of top decision makers’ innovative cognitive style and gender on absorptive capacity.

We used hierarchical regression with robust standard errors to test our results, which are reported in Table 3. Hypothesis 1 predicted that top decision makers’ adaptive cognitive style would be positively related to firm-level AC. As shown in Model 2 of Table 3, this hypothesis was supported (*B* = 0.22, *p* < 0.001). Hypothesis 2 predicted that top decision makers’ innovative cognitive style would be positively related to firm-level AC. This result, also shown in Model 2 of Table 3, indicates that this hypothesis was also supported (*B* = 0.18, *p* < 0.05). Hypothesis 3 predicted that the relationship between top decision makers’ adaptive cognitive style and firm-level AC would be stronger for female top decision makers than for male top decision makers. As shown in Model 3 of Table 3, this hypothesis is supported (*B* = 0.49, *p* < 0.001). The interaction is plotted in Figure 2, which shows the slope of the relationship for women is more positive (simple slope = 0.68, *p* < 0.001) than the slope for men (simple slope = 0.19, *p* < 0.01), supporting the hypothesis. Hypothesis 4 predicted that the relationship between top decision makers’ innovative cognitive style and firm-level AC would be stronger for female top decision makers than for male top decision makers. This hypothesis was supported (*B* = 0.48, *p* < 0.001). The interaction is plotted in Figure 3, which shows the slope of the relationship for women is positive and significant (simple slope = 0.60, *p* < 0.01) whereas the slope for men is not significant (simple slope = 0.12, *p* > 0.05).

**Table 3:** Regression analysis predicting firms’ absorptive capacity.

	Model 1		Model 2		Model 3	
*Predictor variables*	*B*	*Robust SE*	*B*	*Robust SE*	*B*	*Robust SE*
Constant	5.62	4.10	7.95	4.10	7.43	4.09
Top decision maker age	0.01	0.01	0.01	0.01	0.00	0.01
Top decision maker gender	0.25	0.15	0.19	0.14	0.08	0.13
Top decision maker education	0.03	0.05	0.05	0.05	0.06	0.05
Top decision maker founder status	0.27	0.14	0.22	0.14	0.23	0.14
Top decision maker executive experience	0.00	0.01	0.00	0.01	0.00	0.01
Firm size (number of employees)	−0.03	0.03	−0.03	0.03	−0.02	0.03
Firm age	0.00	0.00	0.00	0.00	0.00	0.00
Industry (business-to-business services)	0.25	0.23	0.15	0.21	0.11	0.21
Industry (consumer-oriented services)	0.06	0.23	−0.07	0.21	−0.08	0.21
Adaptive cognitive style (H1)			0.22^***^	0.05	0.19^**^	0.06
Innovative cognitive style (H2)			0.18^**^	0.06	0.12	0.07
Adaptive cognitive style × Top decision maker gender (H3)					0.49^***^	0.13
Innovative cognitive style × Top decision maker gender (H4)					0.48^*^	0.19
*F*	1.79		3.50^***^		6.08^***^	
*R*^2^	0.06		0.13		0.16	

* *p* < 0.05;

** *p* < 0.01;

*** *p* < 0.001;

*N* = 265.

## Discussion

Absorptive capacity refers to a firm’s ability to acquire new knowledge, build knowledge and assimilate information within the firm, and transform knowledge into useful new forms (Cohen and Levinthal, 1990; Zahra and George, 2002). While research on AC has acknowledged the role that individuals, whether employees, managers, or top decision makers may play in fostering firm-level AC (Cohen and Levinthal, 1990; Volberda et al., 2010), studies exploring the individual-level antecedents of firm-level AC have been rare (Distel, 2019). Even less attention has been paid to understanding the relationships between top decision makers and their idiosyncratic attributes and their firms’ AC. Because AC is an important element of firms’ ability to practice strategic entrepreneurship (Ireland et al., 2003) and because top decision makers play a key role in shaping their firms’ behaviors (Hambrick and Mason, 1984), our study may help scholars and practitioners better understand the microfoundations of AC.

We focused on the role that top decision makers’ cognitive styles may play in contributing to their firms’ AC. Cognitive style refers to people’s orientation toward information and how they prefer to act when encountering problems. An adaptive cognitive style leads people to prefer solutions that rely on existing information, that are consistent with others’ expectations, and the adaptive cognitive style is also associated with adherence to established procedures and a high degree of meticulousness (Kirton, 1976). An innovative cognitive style leads people to prefer solutions that are unexpected, that rely on the bisociation of unrelated information, and the innovative cognitive style is also associated with a willingness to violate others’ expectations and abandon previously tested norms and procedures (Kirton, 1976). While early research portrayed these cognitive styles as opposite ends of a single continuum, more recent work has treated them as independent (e.g., Miron-Spektor et al., 2011). AC, too, as elements that resemble these cognitive styles: Cohen and Levinthal (1990) and Zahra and George (2002) emphasize the importance of memory, prior experiences, association, and routines while also valuing newness, whether the newness of information, the newness that flows from the transformation, or newness in the form of creating innovative products and services that AC enables. Our findings – that both adaptive and innovative cognitive styles have positive effects on AC – echoes research that describes the importance of routines and newness, association and bisociation to building and maintaining AC.

Our study contributes to AC research by focusing on the microfoundations of firms’ ability to acquire, assimilate, and transform new information. While early research emphasized the notion that firms develop AC based on the underlying knowledge and cognitive capabilities of the individual people who work in them (Cohen and Levinthal, 1990), there has been little theoretical or empirical work that links individuals’ attributes to firm-level AC (Volberda et al., 2010). Meanwhile, there has been growing scholarly recognition of the nature and importance of the cognitive attributes of firms’ managers and leaders (Kaplan, 2011). In particular, the cognitive and other attributes of firms’ top decision makers have been found to influence firm behaviors and performance (Wang et al., 2016), including how they scan the environment for information concerning their personal and firm performance (Pryor et al., 2019). This paper takes the next step by linking what we know concerning how top decision makers can influence their firms’ pursuit of information with the firm-level capacity to absorb and use new information (AC).

This study also contributes to research on top decision maker attributes and how those attributes affect firm outcomes. In particular, empirical research drawing on upper echelons theory has more recently focused on the psychological and cognitive differences among the top decision makers (Bromily and Rau, 2016; Wang et al., 2016). At the same time, there has been increasing interest in the role that top decision makers and other individuals play in fostering firms’ capabilities (Helfat and Peteraf, 2015). Therefore, given the importance of AC for firm strategy, innovation and performance – and given that it is key to firms’ strategic entrepreneurship – it is surprising that little research has been devoted to understanding the top-decision-maker-AC link.

Relatedly, this study expands our knowledge concerning gender differences of top decision makers and how these differences can influence firm outcomes (Post et al., in press). Empirical evidence suggests that female top decision makers can improve their firms’ financial performance and innovative output (e.g., Lyngsie and Foss, 2017; Hoobler et al., 2018). However, the mechanisms that link top decision maker gender with firm-level outcomes remain murky. While a growing stream of research has focused on developing a finer-grained understanding of these issues, this paper joins a handful of studies that have taken up the issue of executive gender and is among the first to link gender-based cognitive differences among top decision makers to firm-level outcomes (Bromily and Rau, 2016).

Lastly, we contribute to the growing stream of microfoundations research concerning firms’ capabilities and especially AC (Yao and Chang, 2017). While scholars have deeply explored macro and environmental antecedents of AC – research that has significantly developed our understanding of how firms come to possess robust AC – much less emphasis has been devoted to understanding antecedents of AC beneath the firm level. Top decision makers influence their firms’ behaviors *via* several mechanisms, whether intended, such as by setting the strategic trajectory of their firms or by establishing human resource practices that encourage employees to enact desired behaviors (Finkelstein, 1992), or unintended, such as by serving as role models that others in the firm emulate without any explicit prompting from management (Pryor et al., 2015). If firms desire to enact effective AC and if scholars desire to refine their understanding of AC, it could be important to further research these microfoundations (Felin et al., 2012).

Our study also has a number of limitations, which may serve as avenues for further research. First, the cross-sectional nature of our study makes it difficult to assess the direction of causality between top decision makers’ cognitive styles and AC. For instance, it could also be that firms with strong AC are more likely to hire top decision makers who have personal or cognitive attributes that align with their existing competencies. Further research is needed to parse this issue. Second, and related to the cross-sectional nature of our data, we are unable to assess the potential long-term effects of top decision makers’ cognitive styles, which may not always be beneficial for AC. For instance, top decision makers who have an innovative cognitive style do not mind taking risks, breaking existing procedures, or reframing issues in order to solve problems or process new information. While these tendencies may be beneficial for firms’ AC in the short term because it can help foster the acquisition of new knowledge, it may be possible that in the long term, these tendencies may hurt firms ability to build a lasting AC capability, which depends upon existing knowledge and organizational procedures. Third, further research could more thoroughly explore the underlying mechanisms that lead to the gender moderation effects we uncovered. While our theorizing is consistent with extant research on gender and management differences, finer-grained analyses may be able to more firmly establish the mechanisms by which top decision makers’ gender moderate their cognitive styles.

## Data Availability Statement

The raw data supporting the conclusions of this article will be made available by the authors, without undue reservation.

## Ethics Statement

The studies involving human participants were reviewed and approved by OSU Human Subjects Research Office, Oklahoma State University. The patients/participants provided their written informed consent to participate in this study.

## Author Contributions

CP collected the data and developed the first draft. RH and YJ helped in the revision process. All authors contributed to the article and approved the submitted version.

### Conflict of Interest

The authors declare that the research was conducted in the absence of any commercial or financial relationships that could be construed as a potential conflict of interest.

## Footnotes

^1^As a robustness check, we re-ran our analyses using the sem function in Stata 14 with an mlmv estimator to model for missing data, which substantiated the results we report.
